# Genomic compartmentalization of pervasive sex-biased gene expression in the vine mealybug *Planococcus ficus*

**DOI:** 10.1093/g3journal/jkag235

**Published:** 2026-08-20

**Authors:** Dario Cantu, Rosa Figueroa-Balderas, Mark Sisterson, Andrea Minio, Noé Cochetel, Rachel Naegele, Lindsey Burbank

**Affiliations:** Department of Viticulture and Enology, University of California Davis, Davis, CA 95618, United States; Genome Center, University of California Davis, Davis CA 95618, United States; Department of Viticulture and Enology, University of California Davis, Davis, CA 95618, United States; USDA Agricultural Research Service, San Joaquin Valley Agricultural Sciences Center, Parlier, CA 93648, United States; Department of Viticulture and Enology, University of California Davis, Davis, CA 95618, United States; Department of Viticulture and Enology, University of California Davis, Davis, CA 95618, United States; USDA Agricultural Research Service, Sugarbeet and Bean Research Unit, East Lansing, MI 48824, United States; USDA Agricultural Research Service, San Joaquin Valley Agricultural Sciences Center, Parlier, CA 93648, United States

**Keywords:** paternal-genome elimination, sex-biased gene expression, sexual dimorphism, *Planococcus ficus*, genome assembly, secretome, vine mealybug

## Abstract

The vine mealybug, *Planococcus ficus*, is a globally invasive pest of grapevine and a vector of leaf roll viruses. Like other mealybugs, it reproduces through paternal-genome elimination, a sex-determination system that operates without sex chromosomes and is associated with extreme sexual dimorphism. To characterize genome organization and sex-biased expression in this species, we generated a long-read reference genome spanning 369 Mb with 23,489 annotated genes and macrosynteny conserved with the citrus mealybug, *Planococcus citri*. Resequencing of 4 California field individuals provided the first genome-wide baseline of nucleotide diversity for *P. ficus* and 132 cross-validated microsatellite markers for population monitoring. Phloem feeders secrete proteins that mediate host interaction; of 2,129 predicted secreted proteins in *P. ficus*, a conserved core is shared with *P. citri*, and each carries a lineage-specific set. Comparing adult male and female transcriptomes, we found sex-biased expression to be pervasive and skewed toward females: 41% of tested genes differed between the sexes, with female-biased genes both more numerous and showing larger fold changes. These female-biased genes were not randomly distributed but concentrated in discrete blocks of coordinately expressed, tandemly duplicated gene families, a pattern not previously described in a mealybug. Male- and female-biased secreted proteins also differed in origin, with male-biased proteins drawn from a conserved repertoire shared with *P. citri* and female-biased proteins spanning a more lineage-specific pool. Together, these results reveal a female-skewed, spatially clustered architecture of sex-biased expression in a mealybug that lacks sex chromosomes, and provide genomic resources for managing an invasive vineyard pest.

## Teaser

The vine mealybug is an invasive insect that damages vineyards worldwide and spreads grapevine viruses, and it reproduces through an unusual system that determines sex without sex chromosomes. Using a new reference genome Cantu *et al*. built from a single female, the authors compared which genes are active in males versus females. About 40% of genes differed between the sexes, and the female-biased genes were grouped into a few tight co-regulated clusters along the genome rather than scattered. This shows that sex differences in mealybugs are organized in a structured, previously undescribed way, and provides genomic tools for monitoring and managing this pest.

## Introduction

The vine mealybug, *Planococcus ficus* (Signoret) (Hemiptera: Pseudococcidae), is a major pest of grapevine worldwide. Native to the Mediterranean basin, the Middle East, and parts of northern Africa, it has invaded California, Mexico, South America, and southern Africa over the past few decades (Daane et al. 2018; Cocco et al. 2021). Damage is both direct and indirect: heavy infestations reduce vine vigor and contaminate clusters with honeydew and sooty mold, while transmission of grapevine leafroll-associated virus 3 (GLRaV-3) and other ampeloviruses causes the greatest economic losses (Tsai et al. 2008; Tsai et al. 2010). Management relies heavily on a narrow set of insecticide chemistries, raising concerns about resistance, nontarget effects, and sustainability (Mansour et al. 2018; Daane et al. 2020). *P. ficus* overwinters under the bark and moves into the canopy as the season progresses, making timing and coverage of contact treatments difficult (Sisterson and Uchima 2024). Developing alternative control strategies, including RNAi, biocontrol, and resistant rootstocks, requires genomic resources that are still limited for this species.

*P. ficus* belongs to the Pseudococcidae, a family of roughly 2,000 unarmored scale insects within the infraorder Coccomorpha (Hemiptera: Sternorrhyncha) (Williams and Hodgson 2014). Relationships among the major lineages of Coccomorpha have been substantially clarified by recent molecular and genomic phylogenies (Choi and Lee 2022), but divergence times within Pseudococcidae remain less well characterized, with few genome-based estimates for the major pest genera (Fig. 1). The genus *Planococcus* includes several agricultural pests in addition to *P. ficus*, most notably *Planococcus citri* (citrus mealybug) and *Planococcus minor* (passionvine mealybug). *P. ficus* overlaps with *P. citri* in host range, and the 3 species are difficult to separate by morphology alone, often requiring molecular markers for reliable identification (Daane et al. 2011). Genome assemblies are now available for more than a dozen Coccomorpha species, including chromosome-level assemblies for several Pseudococcidae and Coccidae (Li et al. 2020; Ross et al. 2024), providing a comparative framework that until recently was limited to a handful of draft genomes. Population-genetic studies indicate that invasive *P. ficus* populations, including those in California, carry reduced diversity consistent with founder effects (Daane et al. 2018).

**Fig. 1.**
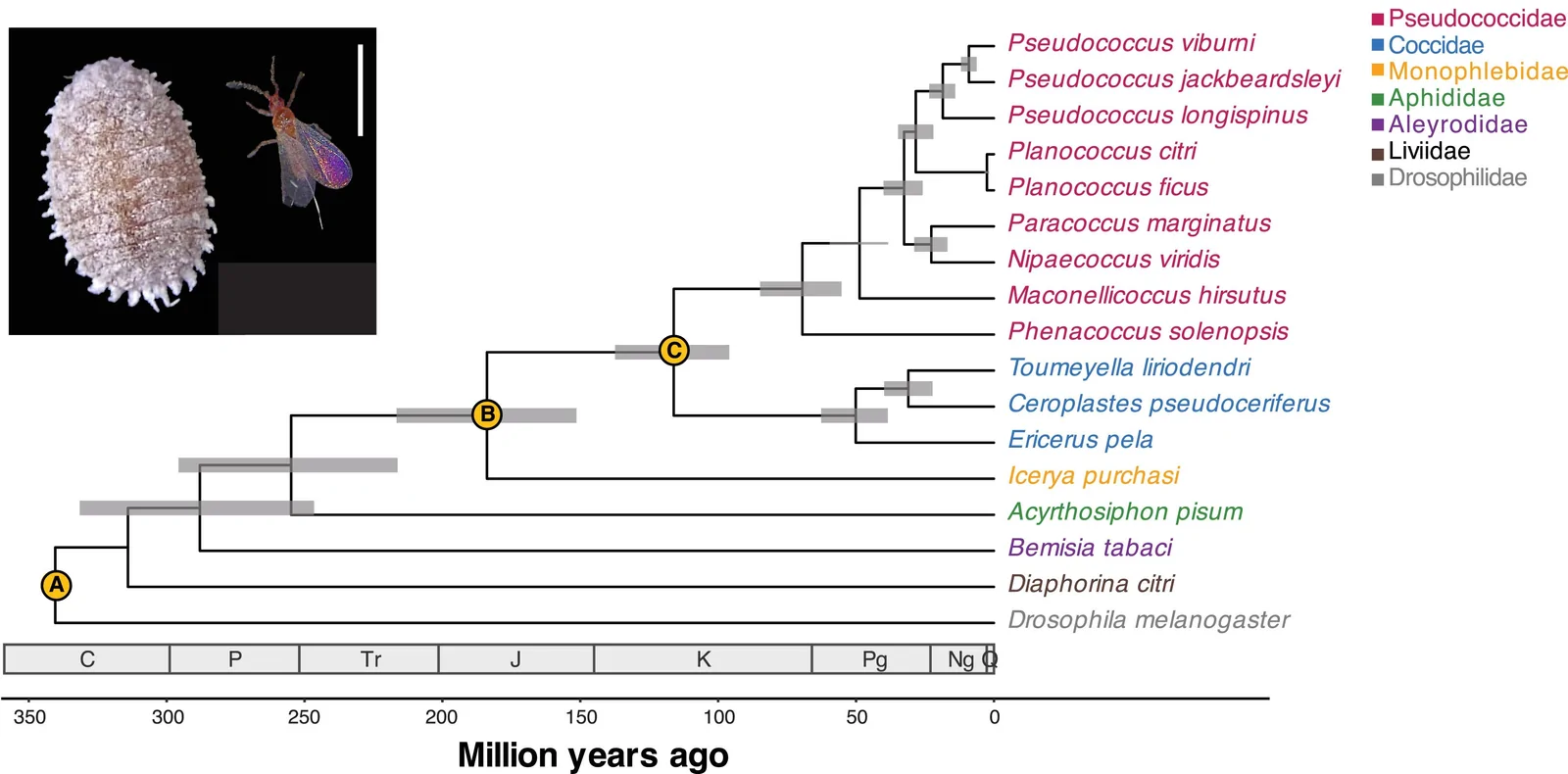
Time-calibrated phylogeny of *P. ficus* (male and female in photo insets, left, and right, respectively; photos by Zachary Dashner) and 16 related Hemiptera, rooted with *D. melanogaster*. Maximum-likelihood topology inferred with IQ-TREE under LG + R4 from a concatenated amino-acid supermatrix; all internal nodes received 100% SH-aLRT and 98% to 100% UFBoot support. Divergence times were estimated with MCMCTree (PAML 4.9j) under an independent-rates relaxed clock, calibrated at 3 fossil-anchored nodes (gold circles): node A, Diptera–Hemiptera split, soft bounds 300 to 365 Ma (Misof et al. 2014); node B, Coccomorpha crown, minimum 130 Ma from *H. patefactus* in Lebanese amber (Vea and Grimaldi 2015); node C, Neococcoidea crown, minimum 99 Ma from *K. perpetuus* and *R. prisca* in Burmese amber (Shi et al. 2012; Vea and Grimaldi 2015). Minimum-age constraints were applied as soft lower bounds with 2.5% tail probability. Gray bars: 95% HPD intervals. Geological periods: C, Carboniferous; P, Permian; Tr, Triassic; J, Jurassic; K, Cretaceous; Pg, Paleogene; Ng, Neogene; Q, Quaternary.

Mealybugs exhibit paternal-genome elimination (PGE), a genetic system in which both sexes are diploid at fertilization but the paternally inherited chromosome set is heterochromatinized and largely transcriptionally silenced in males during early embryogenesis (Kol-Maimon et al. 2014; Bain et al. 2021; de la Filia et al. 2021; Herbette and Ross 2023). The paternal origin of this heterochromatic chromosome set in male mealybugs was established by radiation genetics and cytology in the early 1960s (Brown and Nelson-Rees 1961; Nelson-Rees 1962; Brown and Nur 1964). Males are therefore often described as functionally haploid, although somatic paternal silencing is in practice partial (de la Filia et al. 2021). During a highly modified, inverted meiosis, the paternal chromosomes are partitioned into degenerating spermatids, so males transmit almost exclusively their maternally derived genome, with rare leakage of single paternal chromosomes (Bongiorni et al. 2004; de la Filia et al. 2019). PGE is widespread in arthropods, where it has evolved independently at least 6 times and occurs in over 10,000 species (Gardner and Ross 2014; de la Filia et al. 2015; Herbette and Ross 2023). This reproductive strategy has implications for the evolution of sex-biased gene expression, dosage compensation, and pesticide resistance (Brun et al. 1995; Carrière 2003). Sex-specific transcriptomes of *P. ficus* are expected to reveal how extensively expression differs between the 2 sexes and how that variation is distributed across the genome, providing a framework for interpreting sex-biased expression in a species whose sex arises under PGE rather than through sex chromosomes (Herbette and Ross 2023; Mongue et al. 2026).

Like other phloem-feeding hemiptera, *P. ficus* injects saliva into host tissues during feeding, delivering a complex mixture of proteins and small molecules that modulate plant defenses, alter phloem sieve element function, and facilitate sustained ingestion (Will et al. 2013). In aphids, whiteflies, and psyllids, the salivary secretome includes enzymes that degrade or modify plant cell walls, such as pectinases and cellulases (McAllan and Adams 1961; Ma et al. 1990; Sharma et al. 2014; Silva-Sanzana et al. 2020), calcium-binding proteins that prevent sieve tube occlusion (Will et al. 2007), and small secreted proteins that suppress jasmonate- and salicylate-mediated immunity (Elzinga and Jander 2013); the mealybug *Phenacoccus solenopsis* likewise suppresses host defenses by manipulating jasmonate–salicylate crosstalk (Zhang et al. 2015). The salivary secretome of mealybugs nonetheless remains poorly characterized compared with that of aphids (Carolan et al. 2011), despite the economic importance of the group and its role as a virus vector. Cataloging predicted secreted proteins in the *P. ficus* genome and comparing them to those of *P. citri* and other Coccomorpha will identify conserved core effectors, lineage-specific expansions, and candidates that may underlie host range differences or interactions with grapevine leafroll viruses during transmission (Tsai et al. 2008; Almeida et al. 2013).

Here, we present a long-read reference genome for *P. ficus*, annotate its candidate secreted-protein repertoire in comparison with *P. citri*, benchmark genetic diversity in California populations and develop cross-validated SSR markers, and map sex-biased gene expression genome-wide. We show that sex-biased expression is pervasive and female-skewed that female-biased genes are organized into discrete genomic blocks of coordinately coexpressed tandem paralogs, and that the male- and female-biased secretomes differ in evolutionary character. Together these resources and analyses establish a genomic foundation for *P. ficus* and place its sex-biased expression in the context of PGE.

## Materials and methods

### Biological material

Vine mealybugs came from 2 sources. For reference genome and transcriptome sequencing, insects were drawn from a closed laboratory colony (*P. ficus* VMBCA21f) established in 2015 from a field collection in Selma, California, and reared continuously thereafter on squash as a large population, with no introduction of wild individuals and no intentional bottlenecking. For evaluation of genetic diversity, 4 additional insects (*P. ficus* MB21-2, MB21-3, MB21-P, and MB21-Q) were collected on 2021 August 12 from 4 vineyards in the San Joaquin Valley of California (1 near Parlier, 2 near Jasmin, and 1 near Saco) and used to establish independent colonies. Colonies were maintained on squash following Sisterson and Uchima (2024). To establish colonies from field-collected material, infested grape clusters were transported to the laboratory in a cooler and placed in 60 × 43 × 15 cm trays lined with paper towels together with squash (butternut or acorn); as the clusters dried, mealybugs moved onto the squash. Infested squash were transferred to 40 × 50 × 36 cm storage totes lined with vermiculite to absorb moisture, each tote set on a mat encircled with Stikem Special (Seabright Laboratories, Emeryville, CA) to prevent crawler escape. Colonies were held at room temperature (∼23 °C) under ambient light. Individuals for sequencing were reared as virgins from these colonies. Grape seedlings (∼15 to 20 cm, open-pollinated “Chardonnay”) were placed on colony cages where the mobile first-instar nymphs (crawlers) roamed; once ∼100 crawlers had settled, the seedlings were moved to a growth chamber (27 °C, 14:10 L:D) for 9 to 12 d. The seedlings were then cut and placed in 11 cm deli containers (Amerifoods Trading Company, Los Angeles, CA); as the plant material dried, mealybugs withdrew their mouthparts and began roaming, and individuals were transferred with a fine brush to ∼5 cm grape seedlings and sealed in cages (Sisterson and Uchima 2024). Cages were held for a further 2 weeks (27 °C, 14:10 L:D) until maturity. Because males typically pupate around 14 d under these conditions (Sisterson and Uchima 2024), insects were separated and held individually before reaching reproductive maturity.

### DNA library preparation and sequencing

High-molecular weight genomic DNA from a single female insect was isolated using the Quick DNA HMW MagBead kit (Zymo Research, Irvine, CA, United States). DNA purity, quantity, and integrity were evaluated with a Nanodrop 2000 spectrophotometer (Thermo Scientific, Hanover Park, IL, United States), with the DNA High Sensitivity kit on a Qubit 2.0 Fluorometer (Life Technologies, Carlsbad, CA, United States), and by FEMTO Pulse System (Agilent, Santa Clara, CA, United States), respectively. SMRTbell libraries were prepared following the PacBio Ultra-Low DNA Input Library Preparation using the SMRTbell Express Template Prep Kit 2.0 protocol (PN 101-987-800, Version 01, Pacific Biosciences, Menlo Park, CA, United States). Briefly, 10 ng of HMW gDNA was sheared to a modal size of 10 to 15 kb using Diagenode's Megaruptor (Diagenode LLC, Denville, NJ, United States) and subjected to DNA damage repair and end repair/A-tailing. PCR adapters were then ligated to the repaired fragments, followed by 15 cycles of PCR amplification to generate sufficient material for library construction. The amplified DNA was pooled, subjected to a second round of DNA damage repair and end repair/A-tailing, and converted into SMRTbell libraries through ligation of SMRTbell adapters. Libraries were purified and size-selected using the BluePippin instrument (Sage Science, Beverly, MA, United States) with a size selection range of 8 to 17 kb. The size-selected library was cleaned using 1× ProNex beads (Promega, Madison, WI, United States), and its concentration and final size distribution were evaluated using a Qubit HS Assay Kit (Thermo Scientific) and the Femto Pulse System (Agilent), respectively. The final library was sequenced on a PacBio Sequel IIe (DNA Technology Core Facility, University of California, Davis), yielding 36.8 million continuous long read (CLR) subreads totaling 392.3 Gb (mean subread length: 10.7 kb), corresponding to ∼1,060× raw subread coverage of the primary assembly; overlaps were subsequently capped at 400× during assembly (--max_cov 400). For short-read DNA sequencing, 200 ng of HMW gDNA was used for library preparation using the Kapa LTP library prep kit (Kapa Biosystems, Wilmington, MA, United States). The DNA library was evaluated for quantity and quality with the High Sensitive chip on a Bioanalyzer 2100 (Agilent Technologies) and sequenced in paired-end 150 bp reads on an Illumina HiSeq X Ten instrument (IDseq, CA, United States).

### Reference genome sequencing and assembly

De novo assembly was performed with FALCON and FALCON-Unzip from falcon-kit 1.8.1 (Chin et al. 2016). In the read-correction stage, raw subreads were processed with a seed length cutoff of 100 bp. Pairwise overlaps were detected with daligner (-e0.7 -l100 -k16 -h64 -w7 -s100), tandem and interspersed repeats were masked with HPCTANmask and HPCREPmask (-k18 -h480 -w8 -e.8 -s100; REPmask_code 1,15;2,10;4,10), and error-corrected reads were generated with falcon_sense requiring at least 70% identity and 3× coverage (--min_idt 0.70 --min_cov 3 --max_n_read 400). In the assembly stage, error-corrected reads of at least 7 kb were used as input. Overlaps among corrected reads were computed with daligner (-t60 -k18 -h256 -e.8 -l100 -s100) and filtered with a highly heterozygous setting (--max_diff 100 --max_cov 400 --min_cov 4) to retain divergent allelic contigs, chosen given the heterozygosity range (0.23% to 0.33%) estimated from k-mer analysis. FALCON-Unzip partitioned the assembly into primary contigs (Ps) and haplotigs (Hc). Both haplotype sets were polished with Arrow using gcpp (GenomicConsensus, PacBio). Primary (Ps) contigs were scaffolded with SSPACE-LongRead (Boetzer and Pirovano 2014) using the PacBio subreads (BLASR alignment), with a minimum alignment identity of 70%, a minimum of 10 links, a link ratio of 0.3, and a minimum overlap of 10; haplotigs (Hc) were retained as unscaffolded contigs. The combined Ps + Hc assembly was carried forward for decontamination, annotation, and downstream analyses. Each scaffold was compared by nucleotide BLAST (blastn; NCBI BLAST + 2.7.1) against a custom local database of reference genomes spanning bacteria, archaea, fungi, plants, and metazoans, and the best hit per scaffold was retained and classified by source organism. Scaffolds whose best hits were to nonarthropod genomes with high query coverage and identity were flagged as contamination. Flagged scaffolds, together with their associated gene, transcript, and repeat annotations, were removed prior to all downstream analyses.

Genome size, heterozygosity, and repeat content were estimated from Illumina short reads of 4 California *P. ficus* individuals rather than from the PacBio reads used for assembly because k-mer spectrum analysis requires the low per-base error rate of short reads and is unreliable on CLR reads. K-mers (*k* = 21) were counted with Jellyfish v.2.2.7 (Marçais and Kingsford 2011) and modeled with GenomeScope2 (v2.0; Ranallo-Benavidez et al. 2020) under a diploid model. Assembly statistics were computed with seqkit and samtools. Completeness was assessed with BUSCO v.6.0.0 (Manni et al. 2021) against the hemiptera_odb12 lineage in genome mode (miniprot-based prediction) and protein mode (longest isoform per locus). The *P. ficus* assembly was aligned to the chromosome-level *P. citri* reference (GCF_950023065.1) with minimap2 v.2.28 (Li 2021) using the asm20 preset and --secondary = no. Alignments shorter than 2 kb or on unplaced *P. citri* scaffolds were discarded. Each *P. ficus* scaffold was assigned an orientation from its dominant alignment strand to *P. citri*, and collinear alignments within 1 Mb of one another on both query and target were chained into syntenic blocks. Gene-level synteny was independently assessed with MCScanX (Wang et al. 2012) from DIAMOND v.2.2.0 blastp alignments (e-value 1e-10; Buchfink et al. 2021) between the longest protein isoforms of the 2 species, using default MCScanX parameters (minimum 5 collinear genes per block).

### Gene prediction and functional annotation

Protein-coding genes were predicted with the EVidenceModeler-based pipeline of the Cantu lab (https://github.com/andreaminio/AnnotationPipeline-EVM_based-DClab; Cochetel et al. 2021) over the combined Ps + Hc assembly. *P. ficus* RNA-seq libraries described below were assembled de novo and genome-guided with Trinity v.2.6.5 (Haas et al. 2013) and, after HISAT2 v.2.0.5 alignment (Kim et al. 2015), with StringTie v.1.3.4d (Pertea et al. 2015); coding regions were extracted with TransDecoder v.3.0.1 (TransDecoder). Transcripts were aligned to the assembly with GMAP v.2015-09-29 (Wu and Watanabe 2005), BLAT v.36x2 (Kent 2002), and Magic-BLAST v.1.4.0 (Boratyn et al. 2019), and reconciled into alignment assemblies with PASA v.2.3.3 (Haas et al. 2003). Protein evidence was aligned with exonerate v.2.2.0 (Slater and Birney 2005). A training set derived from the PASA assemblies was used to train AUGUSTUS v.3.0.3 (Stanke et al. 2006), GeneMark.hmm v.3.47 (Lomsadze et al. 2005), and SNAP v.2006-07-28 (Korf 2004). Gene models were predicted with these ab initio predictors, a BUSCO-trained AUGUSTUS model (BUSCO v.3.0.2, Insecta and Eukaryota; Manni et al. 2021), and PASA/TransDecoder-derived models. All prediction tracks, transcript alignments and exonerate protein alignments were integrated with EVidenceModeler v.1.1.1 (Haas et al. 2008), weighting PASA assemblies most heavily (weight 20) and PASA/TransDecoder models (25), followed by ab initio predictions, transcript alignments, and protein alignments. Consensus models were polished and updated for UTRs and alternative isoforms with PASA, then filtered to remove transposable-element and low-confidence models using DIAMOND/BLAST against NCBI RefSeq invertebrate (Camacho et al. 2009). Functional annotation was assigned with Blast2GO v.4.1.9 (Gotz et al. 2008) from DIAMOND/BLAST homology against NCBI RefSeq and InterProScan v.5.28-67.0 (Jones et al. 2014) domain assignments.

### Prediction of secreted proteins

Signal peptides in the longest annotated isoform per gene of *P. ficus* and *P. citri* (NCBI RefSeq GCF_950023065.1; Ross et al. 2024) were predicted with SignalP 6.0 in eukaryotic fast mode (Teufel et al. 2022). Proteins with an SP probability ≥0.7 and a mature peptide length of 30 to 500 amino acids were retained as predicted secreted proteins. Mature peptide length and cysteine content were computed directly from the SignalP-trimmed sequences. The 2 filtered secretomes were combined and clustered jointly. All-versus-all sequence similarity was computed with DIAMOND blastp v2.2.0 (*E* ≤ 1 × 10^−5^; Buchfink et al. 2021), and the resulting hits were clustered with MCL v22-282 (Van Dongen 2008) at inflation *I* = 2.0. Clustering yielded 1,596 groups spanning all 4,312 predicted secreted proteins, including 4 proteins that had no significant similarity to any other sequence and were retained as single-member clusters. Clusters were categorized as shared 1:1, shared with expansion in one or both lineages, or lineage-specific (singleton or family expansion without a cross-species homolog). Protein-domain content of the predicted secreted proteins was annotated with InterProScan v5.70-102.0 (Jones et al. 2014).

### Variant calling and preliminary genetic diversity

Whole-genome resequencing reads from 4 field-collected populations of *P. ficus* (MB21-2, MB21-3, MB21-P, and MB21-Q) were aligned to the *P. ficus* VMBCA21f primary assembly with BWA-MEM v0.7.10 (Li and Durbin 2009; Li 2013) and processed through duplicate marking. Per-sample variants were called with GATK HaplotypeCaller (v4.6.2.0; McKenna et al. 2010) in gVCF mode at diploid ploidy, then joint-genotyped with CombineGVCFs and GenotypeGVCFs. SNPs and indels were split and hard-filtered using the GATK best-practice thresholds for small cohorts (used in place of VQSR) (SNPs: QD < 2, FS > 60, MQ < 40, MQRankSum < −12.5, ReadPosRankSum < −8, SOR > 3; indels: QD < 2, FS > 200, ReadPosRankSum < −20, SOR > 10). To enable callable-site denominators for nucleotide diversity estimation, GenotypeGVCFs was rerun with --include-non-variant-sites to produce an all-sites variant call format (VCF) file; variant records were split, hard-filtered, and concatenated back with the invariant records using bcftools v1.23.1 (Danecek et al. 2021) to yield a filtered all-sites call set. Nucleotide diversity (π) was computed with scikit-allel v1.3.13 (Miles et al. 2024) as the sum of per-site mean pairwise differences over biallelic SNPs, divided by the number of callable sites (positions with genotypes called in ≥3 of 4 samples). Feature-stratified π was computed by restricting the all-sites VCF to browser extensible data (BED) intervals derived from the gene annotation (gene, exon, coding DNA sequence (CDS), intron, 5′ UTR, and 3′ UTR) and from the complement of the gene span against the callable genome (intergenic). Per-sample heterozygous-site counts were obtained with bcftools; heterozygous calls were also intersected with 100 kb nonoverlapping windows using bedtools v2.31.1 to obtain per-window heterozygosity distributions. Runs of homozygosity were estimated with bcftools roh using a uniform default allele frequency (--AF-dflt 0.4, used in lieu of a population allele-frequency panel given the small sample size). Transition/transversion ratios were computed with VCFtools v0.1.15 and cross-validated with bcftools stats.

### Microsatellite marker discovery

SSRs were identified in 5 *P. ficus* assemblies: the reference (VMBCA21f) and 4 whole-genome assemblies of MB21-2, MB21-3, MB21-P, and MB21-Q. Raw reads were quality- and adapter-trimmed with Trimmomatic v0.36 (ILLUMINACLIP with TruSeq3-PE-2 adapters, 2:30:10; LEADING:7; TRAILING:7; SLIDINGWINDOW:10:20; MINLEN:36; Bolger et al. 2014). Trimmed reads were error-corrected with SPAdes BayesHammer (--only-error-correction) and then assembled de novo with SPAdes v3.13.0 (Bankevich et al. 2012) using --dataset corrected.yaml -k 77,97,117,127,137,145 --careful --only-assembler. SSR loci were detected with MISA v2.1 (Beier et al. 2017) using minimum repeat numbers of 6 for dinucleotides and 5 for tri-, tetra-, penta-, and hexanucleotide motifs, a maximum interruption length of 100 bp between compound SSRs, and mononucleotide repeats were excluded. Primer pairs flanking each SSR were designed with Primer3 v2.5.0 (Untergasser et al. 2012) on a 500 bp sequence window centered on each repeat (250 bp flank on each side; primer length 18 to 27 bp, optimum Tm 60 °C with design range 57 to 63 °C, guanine-cytosine (GC) content 40% to 60%, product size 100 to 300 bp). Polymorphic markers were identified by anchoring loci across assemblies through perfect identity of both forward and reverse primer sequences: any primer pair recovered with identical sequences in all 5 assemblies was considered a candidate cross-validated marker and classified as polymorphic when the in silico repeat count differed between at least 2 individuals. Candidates were further filtered for primer pair quality (Primer3 pair penalty ≤5, melting temperature 58 to 62 °C, F-R Tm difference ≤2 °C, GC content 40% to 60%, product size 100 to 300 bp) and restricted to loci showing product-size variation strictly proportional to repeat-count differences.

### RNA-seq and sex-biased gene expression

Total RNA was isolated from 3 biological replicate pools of adult female insects (3 insects per pool) and 3 biological replicate pools of adult male insects (4 insects per pool) using the Quick-RNA Tissue/Insect Microprep Kit (Zymo Research). RNA purity, quantity, and quality were assessed using a NanoDrop 2000 spectrophotometer (Thermo Fisher Scientific, Hanover Park, IL, United States), a Qubit 2.0 Fluorometer with the RNA Broad Range Assay Kit (Life Technologies), and electrophoresis, respectively. RNA-seq libraries were prepared using the Illumina TruSeq RNA Sample Preparation Kit v2 (Illumina, CA, United States) following Illumina’s low-throughput protocol. Quantity and quality of the final libraries were evaluated using a High Sensitivity DNA chip on a Bioanalyzer 2100 instrument (Agilent Technologies) and sequenced in 100-bp single-end mode on an Illumina HiSeq instrument at the DNA Technologies Core Facility, University of California, Davis. Total RNA was extracted, mRNA-enriched, and sequenced on Illumina platforms in single-read mode (R1 reads from paired-end libraries were used). Reads were quality-trimmed with Trimmomatic v0.36 (Bolger et al. 2014), then aligned to the decontaminated *P. ficus* reference genome (Ps + Hc) with HISAT2 v2.1.0 (Kim et al. 2019) using --very-sensitive -k 250, allowing up to 250 alignments per read to account for the haplotype duplication in the combined Ps + Hc assembly. Genome-coordinate alignments were converted to transcript coordinates with UBU v1.2 (sam-xlate) (Mose 2025) using a BED file of mRNA coordinates. Transcript-level abundances were estimated with Salmon v1.5.1 (Patro et al. 2017) in alignment-based mode (--libType U --numBootstraps 100 --seqBias --posBias) against the *P. ficus* transcriptome containing both Ps and Hc transcripts.

Because the reference includes both Ps and Hc transcripts, a subset of transcripts is represented twice in the transcriptome; both haplotype sets were included so that reads from divergent alternate alleles are captured rather than lost to reference bias. Salmon’s EM algorithm distributes reads shared between these copies. Transcript-level quantifications were imported into R with tximport v1.30 (Soneson et al. 2016) and aggregated to the gene level using a tx2gene table; gene-level counts at duplicated loci are conservative with respect to statistical power since reads are split between paralog-like haplotype copies. Differential expression between sexes was tested with DESeq2 v1.42 (Love et al. 2014) with sex as the design variable and female as the reference level. Genes were classified as differentially expressed (DE) at |log2FC| >1 and Benjamini–Hochberg (BH) adjusted *P* < 0.05. No manual minimal-count prefilter was applied; DESeq2's default independent filtering was used. Of the 23,489 primary-assembly genes, 2,758 had zero counts across all libraries, and a further ∼2,400 fell below this threshold (mean normalized count ≈ 1.4), leaving 18,013 genes evaluated for sex-biased expression. Per-scaffold enrichment for sex-biased genes was tested with Fisher’s exact tests, comparing the proportion of F-biased and M-biased genes on each scaffold (≥10 tested genes) against the genome-wide background. Resulting *P*-values were corrected with BH. Gene Ontology (GO) enrichment of F-biased and M-biased gene sets was tested with 1-sided Fisher's exact tests (alternative = greater) against the universe of DE-tested genes carrying InterProScan-derived GO annotations. *P*-values were corrected with BH. To identify protein domains overrepresented among sex-biased predicted secreted proteins, each InterPro accession was tested with a 1-sided Fisher’s exact test for enrichment in the focal class (F-biased or M-biased) relative to all other DESeq2-tested predicted secreted proteins. *P*-values were adjusted across all tested domains by the BH procedure.

### Phylogenomic analysis and divergence-time estimation

To place *P. ficus* in a broader phylogenetic context, we compiled a 17-taxon dataset comprising the *P. ficus* VMBCA21f genome assembled in this study, 8 additional Pseudococcidae (*P. citri*, GCF_950023065.1, Ross et al. 2024; *Pseudococcus jackbeardsleyi*, GCA_038380155.1, Guo et al. 2024; *Pseudococcus viburni*, GCA_033439095.1, Vea et al. 2025; *Pseudococcus longispinus*, GCA_900064475.1; *Maconellicoccus hirsutus*, GCA_003261595.1, Kohli et al. 2021; *Ph. solenopsis*, GCA_009761765.1, Li et al. 2020; *Nipaecoccus viridis*, GCA_052327235.1, Liesenfelt et al. 2025; *Paracoccus marginatus*, GCA_048836485.1, Wei et al. 2025), 3 Coccidae (*Ceroplastes pseudoceriferus*, GCA_050872025.1, Qin et al. 2025; *Toumeyella liriodendri*, GCA_041937245.1, Mongue et al. 2024; *Ericerus pela*, GCA_011428145.1), 1 Monophlebidae (*Icerya purchasi*, GCA_039619475.1, Deng et al. 2024), 1 Aphididae (*Acyrthosiphon pisum*, GCF_005508785.2, Li et al. 2019), 1 Aleyrodidae (*Bemisia tabaci*, GCF_918797505.1), 1 Liviidae (*Diaphorina citri*, GCA_024506315.3, Shippy et al. 2024), and *Drosophila melanogaster* (GCF_000001215.4, Hoskins et al. 2015) as a distant outgroup. Single-copy orthologs were identified in each genome with BUSCO v6.0.0 (Manni et al. 2021) against the hemiptera_odb12 database. Per-ortholog protein alignments were generated with MAFFT v7 (--auto; Katoh and Standley 2013), trimmed with trimAl v1.5 (-automated1; Capella-Gutiérrez et al. 2009), and concatenated with AMAS (Borowiec 2016) into a 1.6 Mb amino-acid supermatrix. Maximum-likelihood inference was performed with IQ-TREE v3 (Minh et al. 2020) under the LG + R4 substitution model, with 1,000 ultrafast bootstrap and 1,000 SH-aLRT replicates. All internal nodes received 100% SH-aLRT support and 98% to 100% UFBoot support. Divergence times were estimated with MCMCTree (PAML 4.9j; Yang 2007) using the approximate likelihood method (dos Reis and Yang 2011). The supermatrix was randomly subsampled to 200,000 amino-acid sites (seed 42) to reduce computational requirements. Branch lengths, gradient, and Hessian were estimated under the LG amino-acid rate matrix with discrete Γ rate variation (α = 0.5, 4 categories). MCMCTree was then run under the independent-rates relaxed clock model (clock = 2), with a birth–death-sampling prior on internal node times (parameters 1, 1, 0.1), a Γ(2, 20) prior on the mean substitution rate, and a Γ(1, 10) prior on the rate variance. Three fossil-based node calibrations were applied: (i) the root, representing the Hemiptera–Diptera divergence, was constrained with soft bounds at 300 to 365 Ma based on Carboniferous insect fossils (Misof et al. 2014); (ii) the Coccomorpha crown (*I. purchasi* + Neococcoidea) was given a minimum age of 130 Ma based on *Hodgsonicoccus patefactus* in Lebanese amber (Vea and Grimaldi 2015); (iii) the Neococcoidea crown (Coccidae + Pseudococcidae) was given a minimum age of 99 Ma based on *Kozarius perpetuus* and *Rosahendersonia prisca* in mid-Cretaceous Burmese amber (Vea and Grimaldi 2015; amber U–Pb zircon age 98.79 ± 0.62 Ma, Shi et al. 2012). Minimum-age constraints were implemented as soft lower bounds with the default 2.5% tail probability. Two independent MCMC chains were run from different random seeds with 500,000 burn-in generations followed by 25,000 samples taken every 200 generations. Cross-chain agreement was assessed by comparison of posterior means across all internal nodes; the maximum difference between chains was 1.56%. The 2 chains were pooled (after a further 10% within-chain burn-in) for the final posterior summary.

## Results

### Genome assembly of *P. ficus* VMBCA21f

A reference genome was assembled for a single adult female *P. ficus* (sample VMBCA21f), sequenced with PacBio CLR. Four additional individuals from separate field-collected populations (MB21-2, MB21-3, MB21-P, and MB21-Q) were sequenced with Illumina paired-end reads for resequencing. Six RNA-seq libraries (3 females and 3 males) were generated for gene annotation and sex-biased expression analysis. Haploid genome size was estimated by k-mer (*k* = 21) frequency analysis of the 4 Illumina samples with Jellyfish 2.2.7 and GenomeScope 2.0. Estimates ranged from 386 to 423 Mb, with heterozygosity between 0.23% and 0.33% and repeat content between 28% and 34% (Supplementary Table 1). The unique sequence component was stable across samples (∼278 to 279 Mb), indicating that inter-sample size variation reflects differences in repetitive content rather than gene-space expansion. These estimates are broadly consistent with the ∼370 Mb primary assembly span (Ps; below).

PacBio CLR reads from a single female insect (VMBCA21f) were assembled with FALCON-Unzip, producing 1,755 primary contigs (N50 = 356 kb, 368.6 Mb) and 4,072 alternative haplotigs (N50 = 39 kb, 127.0 Mb). After Arrow polishing, the primary contigs were scaffolded with SSPACE, reducing the number of sequences from 1,755 to 1,323 and increasing the N50 from 356 to 485 kb; haplotigs were retained as Arrow-polished contigs without further scaffolding. The assembly was screened for nonhost sequence by per-scaffold blastn against a custom local database of reference genomes spanning bacteria, archaea, fungi, plants, and metazoans. Four sequences, 3 primary scaffolds, and 1 haplotig (573 kb, 0.12% of the assembly), were identified as contamination and removed: 2 showed end-to-end identity to *Candidatus Moranella endobia* (NC_021057.1, NC_015735.1; query coverage 87% to 95%, identity >92%), the γ-proteobacterial endosymbiont nested inside *Tremblaya princeps* in Pseudococcidae, while 2 additional scaffolds were identified as fungal contamination.

The decontaminated v1 reference comprises a primary assembly (Ps) of 1,320 scaffolds spanning 369.4 Mb (N50 = 484.9 kb, L50 = 239), and an associated set of 4,071 alternative haplotigs (Hc) spanning 127.0 Mb (N50 = 39.1 kb; Table 1; Supplementary Table 2). The Ps assembly contains 40 scaffolds longer than 1 Mb (largest 2.68 Mb) and 931 scaffolds longer than 100 kb. GC content is uniform across partitions (Ps 34.5%, Hc 34.6%); gaps comprise 0.37% of Ps and are absent from Hc.

**Table 1.** Reference assembly statistics for *P. ficus* VMBCA21f.

Metric	Primary (Ps)	Haplotigs (Hc)
Scaffolds	1,320	4,071
Total length (Mb)	369.4	127.0
Largest scaffold (kb)	2,677.8	307.0
Scaffold N50 (kb)	484.9	39.1
L50	239	912
GC content (%)	34.5	34.6
Gap content (%)	0.37	0.00
Repeat-masked (%)	32.0	34.4
Genes	23,489	7,591
mRNAs	33,259	10,374
Median protein length (aa)	382	349
BUSCO genome C[S/D]/F/M (%)^a^	90.0 [82.6/7.4]/1.7/8.3	28.9 [28.2/0.7]/3.3/67.8
BUSCO protein C[S/D]/F/M (%)^a^	86.1 [79.1/6.9]/2.9/11.0	25.9 [25.1/0.8]/3.3/70.8

^a^C, complete; S, single-copy; D, duplicated; F, fragmented; M, missing.

Whole-genome alignment of the *P. ficus* assembly to the chromosome-level *P. citri* reference (GCF_950023065.1; Ross et al. 2024) supported the structural integrity of the assembly: 240 Mb of *P. ficus* sequence (65% of the assembly) aligned to the 5 *P. citri* chromosomes via minimap2, and gene-level synteny analysis with MCScanX recovered 619 cross-species collinear blocks containing 9,123 orthologous gene pairs (Tang et al. 2008; Wang et al. 2012). The 532 large *P. ficus* scaffolds (≥100 kb aligned) mapped uniquely and contiguously to 1 of the 5 *P. citri* chromosomes (Supplementary Fig. 1), with no evidence of inter-chromosomal mis-joins, consistent with conserved macrosynteny across ∼2.5 Ma of *Planococcus* divergence.

Gene prediction was performed on the decontaminated assembly with a combined evidence pipeline integrating ab initio predictions, spliced alignment of RNA-seq evidence assembled with Trinity and StringTie, and protein homology, consolidated by EVidenceModeler and refined by PASA. The Ps annotation contains 23,489 protein-coding genes producing 33,259 transcripts (mean 1.42 isoforms per gene); the Hc annotation contains 7,591 genes and 10,374 transcripts (1.37 isoforms per gene) (Table 1). Repeat annotation identified 549,560 features covering 118.0 Mb of nonoverlapping sequence on Ps (32.0%) and 204,313 features covering 43.7 Mb on Hc (34.4%; Supplementary Table 3). Both partitions are dominated by unclassified repeats (Ps 21.4%, Hc 22.8%), followed by DNA transposons (Ps 5.5%, Hc 5.9%), LTR retroelements (Ps 3.0%, Hc 3.3%), and rolling-circle Helitron elements (Ps 1.8%, Hc 2.1%); LINEs, SINEs, satellites, and simple/low-complexity sequence together contribute the remaining ∼2.4% in each partition (Supplementary Table 3). The high proportion of unclassified repeats reflects the limited prior characterization of repeat families in Pseudococcidae and the use of a de novo repeat library generated for this assembly.

Completeness was assessed with BUSCO v6.0.0 against the hemiptera_odb12 lineage (*n* = 3,396; Supplementary Table 4). The primary assembly recovered 90.0% complete BUSCOs in genome mode and 86.1% in protein mode (longest isoform per gene). The haplotigs recovered substantially fewer markers in either mode (28.9% genome, 25.9% protein), consistent with their partial coverage of the genome as alternative haplotypes.

### The *P. ficus* predicted secreted-protein repertoire

From the 23,489-protein *P. ficus* primary-scaffold proteome (longest isoform per gene), SignalP 6.0 predicted a signal peptide for 3,062 proteins; 2,901 of these had an SP probability ≥0.7. Restricting to a mature peptide length of 30 to 500 amino acids retained 2,129 predicted secreted proteins (9.1% of the proteome) for downstream analyses (Supplementary Table 5). The mature peptides spanned a wide size range (median 248 aa, interquartile range (IQR) 156 to 364). Cysteine content was modest overall (median 1.64%, IQR 0.68% to 2.91%), with only 333 candidates (15.6%) reaching the ≥4% cysteine threshold conventionally used to delimit the classical disulfide-stabilized small Cys-rich secreted-protein class. The *P. ficus* predicted secreted-protein repertoire is therefore dominated by proteins outside this canonical category, which is expected for an insect, since the small cysteine-rich class is the effector archetype of filamentous plant pathogens (fungi and oomycetes) rather than of insects, whose secretomes are functionally diverse (Carolan et al. 2011; Boulain et al. 2018). InterProScan recovered at least one InterPro domain in 1,264 of the 2,129 candidates (Fig. 2a; 59.4%); the remaining 865 (40.6%) had no detectable InterPro signature, indicating a large reservoir of small, possibly fast-evolving secreted proteins whose function cannot be inferred from sequence alone. Within the annotated subset, the most abundant functional groups (Fig. 2b; Table 2) fell into 3 broad groups. Hydrolytic and digestive enzymes were prominent, including S1 chymotrypsin/trypsin-like serine proteases (*n* = 50), C1A papain-like cysteine peptidases of the cathepsin family (*n* = 43), α/β hydrolase-fold enzymes (*n* = 64), triacylglycerol lipases (*n* = 27), UDP-glycosyltransferases (UGTs; *n* = 45), and carbonic anhydrases (*n* = 32); a serpin family (*n* = 30) likely provides negative regulation of the serine protease component. Binding and recognition proteins formed a second group, headed by immunoglobulin-like-fold proteins (*n* = 76), pheromone/general odorant-binding proteins (PBP/GOBP) (*n* = 60), phosphatidylethanolamine-binding (PEBP)-like proteins (*n* = 46), annexins (*n* = 26), and juvenile-hormone-binding/takeout proteins (*n* = 21). A third group of cuticle-associated proteins (*n* = 43) and leucine-rich repeat proteins (*n* = 34) accounted for additional structural and recognition functions. This composition is that of a broad predicted secretome spanning hydrolytic and regulatory enzymes, binding and recognition proteins, and structural families. Several of these are plausibly involved in phloem feeding, but many are likely ordinary secreted or membrane-associated proteins rather than effectors, and we do not interpret the set as a feeding-specific arsenal.

**Fig. 2.**
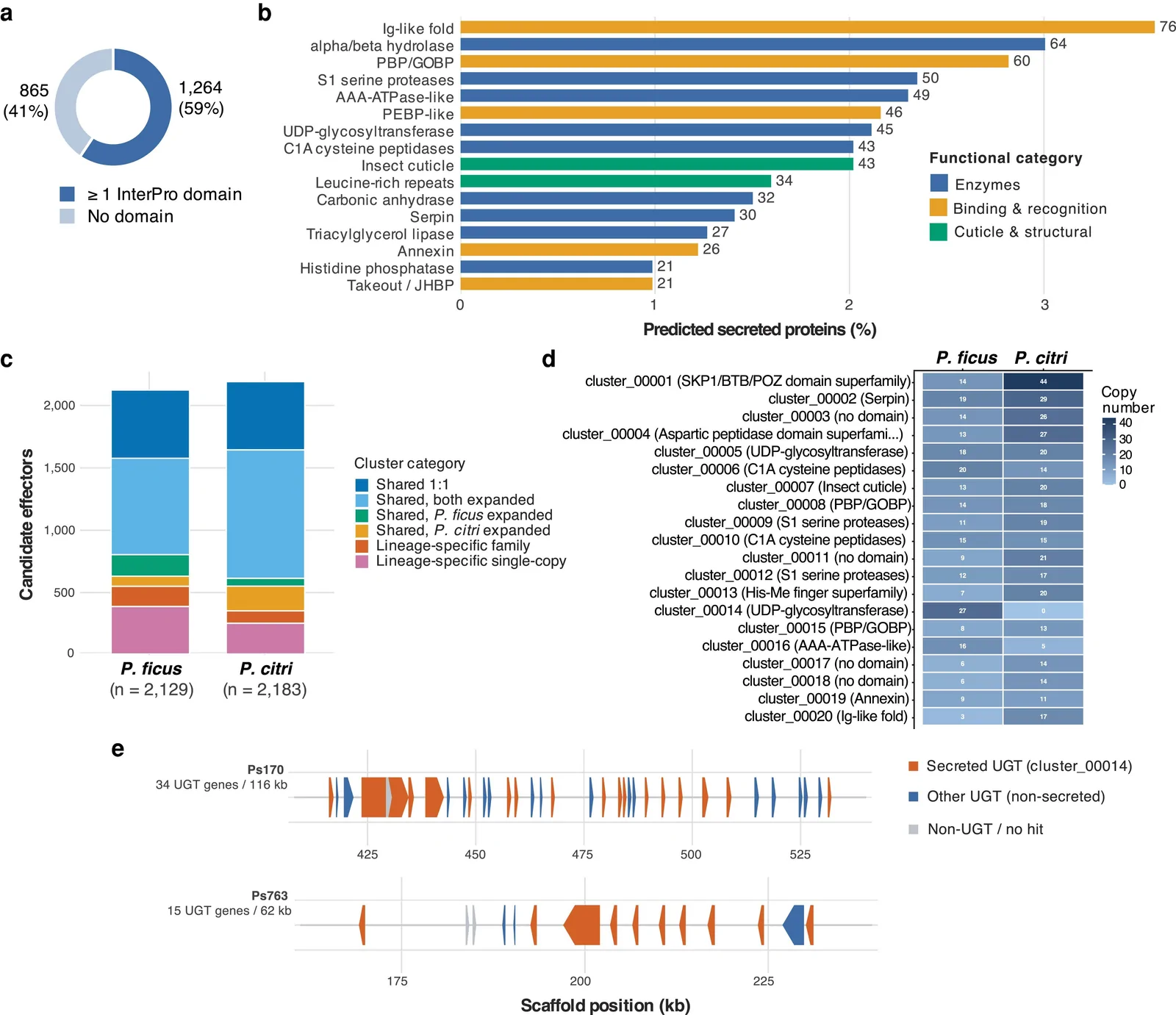
Predicted secreted-protein repertoire of *P. ficus* and cross-species comparison with *P. citri*. a) InterPro domain annotation coverage of the 2,129 predicted secreted proteins. b) Major InterPro functional groups among the 2,129 *P. ficus* predicted secreted proteins, shown as the percentage carrying at least 1 domain in each group (see Table 2). c) Composition of the jointly clustered secretomes of *P. ficus* (*n* = 2,129) and *P. citri* (*n* = 2,183), partitioned by cluster category. d) The 20 most abundant secreted-protein clusters, showing per-species copy number. e) Genomic organization of the *P. ficus*-specific UDP-glycosyltransferase expansion (cluster_00014). Two tandem arrays, on scaffolds Ps170 (34 UGT genes, ∼116 kb) and Ps763 (15 UGT genes, ∼62 kb), are drawn to scale with genes as true-width rectangles and a terminal tick indicating strand.

**Table 2.** Major InterPro functional groups among *P. ficus* predicted secreted proteins.

Category	Functional group	Likely role	Unique proteins	%
Binding and recognition	Immunoglobulin-like fold (Ig-like superfamily)	Cell adhesion, recognition, binding	76	3.57
Enzymes	α/β hydrolase-fold	Broad hydrolase scaffold (esterases, lipases, peptidases)	64	3.01
Binding and recognition	Pheromone/general odorant-binding protein (PBP/GOBP)	Chemoreception, small-molecule transport	60	2.82
Enzymes	S1 serine proteases (chymotrypsin/trypsin-like)	Extracellular proteolysis, digestion	50	2.35
Enzymes	AAA-ATPase-like	ATP-dependent remodeling/chaperone activity	49	2.30
Binding and recognition	PEBP-like	Phosphatidylethanolamine binding; signaling regulation	46	2.16
Enzymes	UDP-glycosyltransferases	Glycosylation of small molecules; xenobiotic detoxification	45	2.11
Enzymes	C1A papain-like cysteine peptidases (cathepsins)	Lysosomal/extracellular protein degradation	43	2.02
Cuticle and structural	Insect cuticle proteins	Cuticle structure and assembly (R&R-type chitin-binding)	43	2.02
Cuticle and structural	Leucine-rich repeats	Protein–protein interaction, immune recognition	34	1.60
Enzymes	Carbonic anhydrases	CO_2_/HCO_3_^−^ interconversion; pH and ion homeostasis	32	1.50
Enzymes	Serpins (serine protease inhibitors)	Regulation of protease cascades; immunity	30	1.41
Enzymes	Triacylglycerol lipases	Lipid hydrolysis	27	1.27
Binding and recognition	Annexins	Calcium/phospholipid binding; membrane trafficking	26	1.22
Enzymes	Histidine phosphatases	Phosphate metabolism	21	0.99
Binding and recognition	Juvenile-hormone binding (takeout/JHBP)	Hormone transport; insect development and behavior	21	0.99

Functional groups defined by collapsing related InterPro accessions within the same family or superfamily and assigned to 3 broad categories (enzymes; binding and recognition; cuticle and structural). Counts are unique proteins per group among the 2,129 *P. ficus* predicted secreted proteins.

The combined *P. ficus* and *P. citri* secretomes were jointly clustered with MCL to assess secreted-protein conservation between the 2 species. The 4,312 predicted secreted proteins partitioned into 1,596 clusters (Fig. 2c; Supplementary Tables 7 and 8). Most predicted secreted proteins of both species belonged to shared clusters with cross-species homologs: 74% of *P. ficus* secreted proteins (1,573/2,129) and 84% of *P. citri* secreted proteins (1,837/2,183; Supplementary Table 6) were assigned to a cluster containing members of both species. Shared single-copy orthologs (547 clusters; 1:1 between the 2 species) represented the deepest core of conserved secreted proteins. Lineage-specific clusters were also abundant, but their counts were asymmetric: *P. ficus* had 556 lineage-specific secreted proteins (381 singletons + 175 in family expansions), compared with only 346 in *P. citri* (247 + 99). The 2 species therefore share a substantial conserved secreted-protein core but differ in the size of their lineage-specific repertoires, with *P. ficus* harboring more novel and expanded private secreted-protein families.

The 20 most populous clusters together accounted for 618 predicted secreted proteins (14.3% of the candidate set) and were dominated by a small number of recurring functional categories (Fig. 2d; Supplementary Table 7). Detoxification and digestive enzymes were prominent: 2 clusters of UDP-glycosyltransferases (clusters 5 and 14), 2 clusters of C1A papain-like cysteine peptidases (clusters 6 and 10), and 2 clusters of S1 serine proteases (clusters 9 and 12) together comprised 6 of the top 20 expansions. Protease regulation appeared as the second most populous cluster overall (cluster_00002, a 48-member serpin family). Chemoreception was represented by 2 PBP/GOBP expansions (clusters 8 and 15). The largest single cluster (cluster_00001, 58 members) was annotated as SKP1/BTB/POZ domain proteins, which most commonly serve as ubiquitin-ligase adaptors; whether the cluster represents a bona fide secreted variant or a misclassified intracellular family in both genomes requires further validation. This warning is specific to this cluster because SKP1/BTB/POZ is a canonical intracellular family, so its predicted secretion is more likely a misassigned N-terminal hydrophobic segment than a genuine signal peptide, and it does not extend to the pipeline as a whole. Confidence in the remaining clusters rests on their dominant annotations corresponding to families with established extracellular or secreted roles; the ATPase Associated with diverse cellular Activities (AAA-ATPase) cluster (cluster_00016) is a comparable exception, its domain being typically intracellular.

Of the 20 most populous clusters, 4 had no detectable InterPro domain (clusters 3, 11, 17, 18; jointly 110 proteins), representing a substantial reservoir of conserved but functionally uncharacterized cross-species secreted-protein families. Several clusters showed pronounced asymmetric expansion between species. The 2 UDP-glycosyltransferase clusters are distinct subfamilies that did not merge at this inflation and share only the broad UGT category: cluster_00005 (18 *P. ficus*, 20 *P. citri*) is a conserved family present at similar copy number in both species and belongs to the shared ancestral UGT core, whereas cluster_00014 (27 *P. ficus*, 0 *P. citri*) is *P. ficus*-specific and is the lineage-specific expansion we focus on here. Its 27 secreted members are the signal-peptide-bearing subset of 2 larger *P. ficus*-specific UDP-glycosyltransferase tandem arrays, comprising 34 genes on scaffold Ps170 (∼116 kb) and 15 on Ps763 (∼62 kb); within each array the secreted and nonsecreted UGTs are interspersed and uniformly oriented, consistent with tandem duplication rather than an assembly artifact (Fig. 2e). cluster_00016 (AAA-ATPase-like; 16 Pfic vs 5 Pcit) represents a more modest *P. ficus*-biased expansion. Conversely, cluster_00013 (His-Me finger nuclease superfamily; 7 Pfic vs 20 Pcit) and cluster_00020 (Ig-like fold; 3 Pfic vs 17 Pcit) are expanded in *P. citri*. These lineage-specific expansions are consistent with divergent host-adaptation pressures between the 2 species, although this interpretation rests on domain annotation and copy number alone; the *P. ficus*-specific UGT expansion is of interest given the reported role of UGTs in xenobiotic detoxification of plant secondary metabolites.

### Diversity and microsatellite discovery

The 4 insects (MB21-2, MB21-3, MB21-P, and MB21-Q), collected from 4 vineyards in California's San Joaquin Valley, were sequenced to depths of 65 to 82 million read pairs each (151 bp paired-end), yielding 49 to 61× mean coverage of the primary assembly. Mapping rates were consistently high across all 4 samples, ranging from 95.8% to 96.3% mapped reads, with 87.8% to 90.3% in proper pairs (Supplementary Table 9). Joint genotyping produced 2,247,614 raw variants. After excluding 3 contaminating scaffolds and applying GATK hard filters, the final call set comprised 1,232,504 SNPs and 517,359 indels (PASS rates 73.6% and 92.6%, respectively). Per-individual coverage at variant sites ranged from 35.8× to 46.7×. Each individual carried 558,223 to 682,850 heterozygous sites, with a mean of 127 to 156 heterozygous sites per 100 kb window. Genome-wide nucleotide diversity, estimated from the all-sites VCF using callable-site denominators, was π = 2.06 × 10^−3^ over 337.2 Mb of callable genome.

Feature-stratified analysis showed that intergenic regions had the highest π (2.09 × 10^−3^), followed by introns (2.03 × 10^−3^) and coding sequence (2.02 × 10^−3^), with the 2 UTR classes substantially depleted (5′ UTR: 1.40 × 10^−3^; 3′ UTR: 1.41 × 10^−3^; Supplementary Table 10). SNP density per kb showed a similar UTR depletion (5′ UTR: 2.33; 3′ UTR: 2.11) but was elevated in CDS (3.75) relative to intergenic (3.33); this elevation is attributable to reduced mappability in repeat-rich intergenic sequence and is not seen in π estimated against the callable denominator. The Ts/Tv ratio was 1.72 genome-wide, rose to 2.37 in coding sequence, and was lower in introns (1.63) and intergenic regions (1.69), consistent with selection against nonsynonymous transversions in coding regions. Ts/Tv was stable across allele-count classes (1.68 to 1.78), across variant-quality bins, and outside repeat-annotated regions (1.76). bcftools-derived Ts/Tv (1.70 to 1.71) agreed with VCFtools to within 0.02 (Supplementary Table 11).

To enable marker development for population studies, each of the 4 individuals was additionally assembled de novo with SPAdes and screened for SSRs alongside the chromosome-level reference. MISA recovered 21,088 to 21,384 perfect SSR loci per assembly (Supplementary Table 12). Cross-assembly anchoring by exact forward and reverse primer identity yielded 953 loci independently recovered in all 5 assemblies: 718 were monomorphic, and 235 showed repeat-count variation between individuals. Filtering for primer quality and strict repeat-proportional product-size variation retained 132 cross-validated polymorphic SSR markers (Supplementary Table 13).

### Pervasive sex-biased expression

The 6 adult RNA-seq libraries (3 females and 3 males) comprised 41.8 to 79.8 million single-end reads each, of which 83.6% to 89.7% mapped to the *P. ficus* reference genome with HISAT2. Sex-biased expression was pervasive. Among the 18,013 Ps host genes tested with DESeq2, 7,362 (40.9%) were significantly DE between sexes (|log_2_FC| >1, BH-adjusted *P* < 0.05; Supplementary Table 14): 4,500 F-biased and 2,862 M-biased on the Ps partition (Fig. 3, a and b), with a comparable but smaller bias on the haplotig partition (1,578 F-biased, 876 M-biased; Supplementary Fig. 2, a and b). At the genome-wide level, the median log_2_FC across all expressed Ps genes was −0.33, shifted toward female bias but falling short of the −1 expected under naive halving of paternal alleles in males (Fig. 3c). This is consistent with the incomplete somatic silencing reported in *P. citri* (de la Filia et al. 2021), though bulk RNA-seq cannot distinguish residual paternal expression from active dosage compensation.

**Fig. 3.**
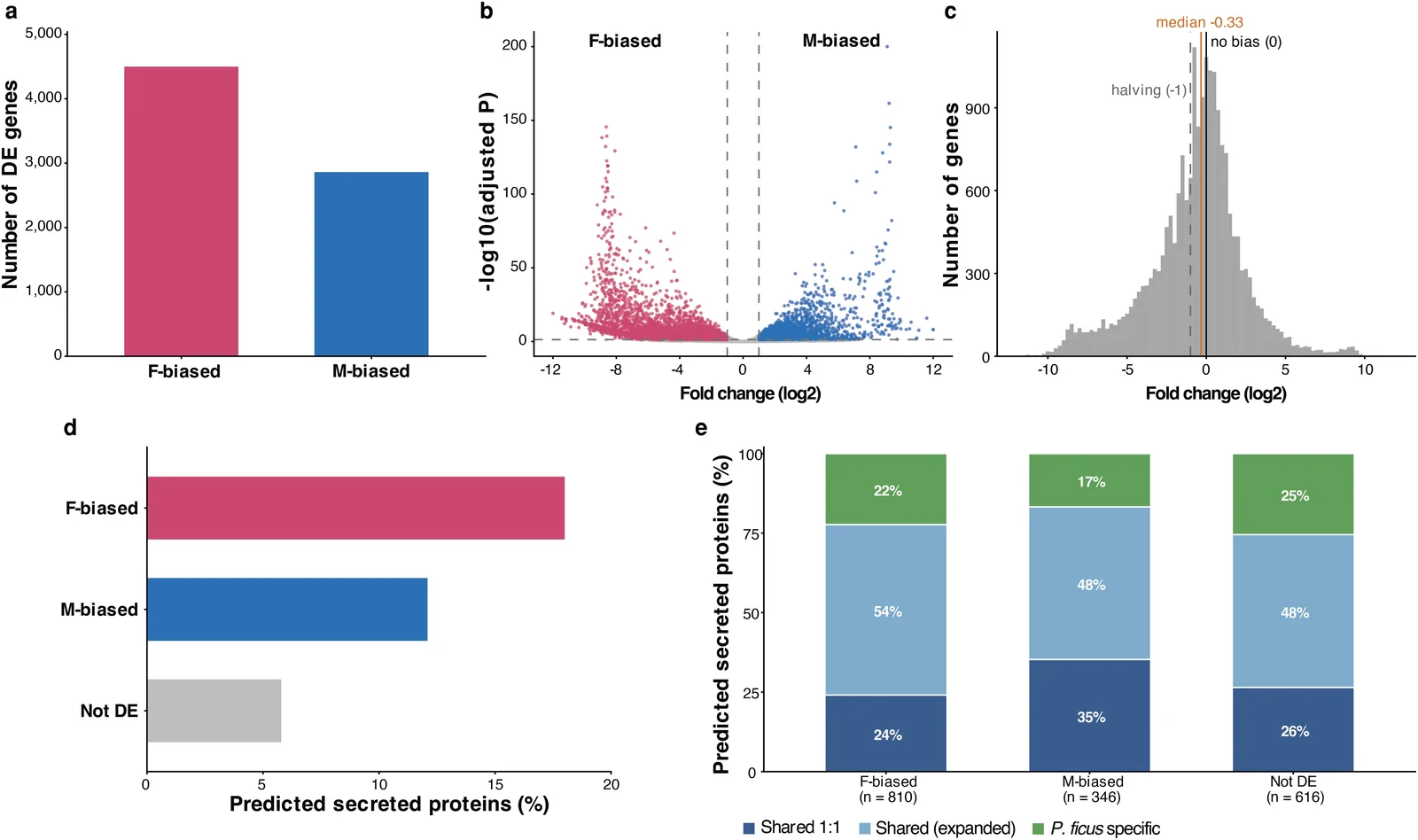
Sex-biased gene expression and the secreted-protein repertoire in *P. ficus*. Differential expression between adult males and females across the primary-scaffold (Ps) gene set, with female as the reference (log_2_ fold change = M/F); sex-biased at |log_2_FC| >1 and adjusted *P* < 0.05 (BH). a) Counts of sex-biased Ps genes by direction. b) Volcano plot of log_2_FC against significance; dashed lines mark the |log_2_FC| = 1 and *P* = 0.05 thresholds. c) Distribution of log_2_FC across all Ps loci, with the observed median (orange), no-bias expectation (0, black), and naive paternal-halving prediction (−1, dashed gray) marked. d) Predicted secreted proteins (SignalP 6.0, SP probability ≥0.7, mature peptide 30 to 500 aa) as a percentage of Ps genes in each DE class. e) Predicted secreted proteins by DE class and cross-species conservation, scored against *P. citri* orthologous clusters as shared 1:1, shared with family expansion, or *P. ficus*-specific; *n* below each bar is the number of predicted secreted proteins with cluster assignments.

Functional enrichment (Supplementary Fig. 3 and Table 15) identified distinct sex-specific programs. Male-biased genes were enriched for the locomotor and sensory machinery of dispersing adult males: odorant binding (*n* = 49, fold enrichment 3.1, *P*_adj_ = 1.1 × 10^−13^, the strongest male-biased term), nervous system process (*n* = 120, 2.0×), muscle system process, cilium and synapse organization, and ion transport. Female-biased genes were enriched for translation and ribosome biogenesis (*n* = 203, 2.4×, *P*_adj_ = 1.4 × 10^−38^, the most significant term overall), mRNA metabolism, and nuclear/nucleolar components, alongside more specific categories including negative regulation of the ERK1/2 cascade (4.5×), carboxylic ester hydrolase activity (*n* = 107, 2.7×), antimicrobial defense, DNA repair, and gene silencing by RNA.

Given the female-specific feeding behavior of *P. ficus*, we asked whether the candidate secreted proteins identified above showed a corresponding female bias. F-biased genes were strongly enriched for predicted secretion (18.0%) compared with M-biased genes (12.1%) and the nondifferentially expressed background (5.8%); the F-biased Fisher's odds ratio (OR) of 2.86 (*P* = 3 × 10^−89^) was over twice that of M-biased (OR 1.32, *P* = 1.6 × 10^−5^; Fig. 3d). Stratifying these predicted secreted proteins by their cross-species cluster category revealed that F-biased secreted proteins tracked the global *P. ficus* secreted-protein distribution, whereas M-biased secreted proteins were significantly enriched in deeply conserved shared 1:1 orthologs with *P. citri* relative to F-biased secreted proteins (35% vs 24%; Fisher's exact test, OR 1.72, *P* = 1.3 × 10^−4^; Fig. 3e). The 2 sex-biased secretomes therefore differ in the conservation of their members: the male-biased set is drawn preferentially from an ancestral, conserved repertoire, while the female-biased set spans the more lineage-specific *P. ficus* secreted-protein pool.

Among M-biased secreted proteins, 8 InterPro domains were significantly enriched (Table 3), with 2 clear functional themes. Male-specific chemoreception was the dominant signal: PBP/GOBP (IPR036728 and IPR006170) reached the strongest enrichment of the analysis (OR 6.2 and 5.7, BH-adjusted *P* = 9.2 × 10^−8^ and 6.6 × 10^−6^). Adult cuticle and exoskeleton biology was the second: 3 insect cuticle protein families (IPR000618, IPR050468, and IPR051217), and the chitin-binding R&R consensus motif (IPR031311) were all enriched among M-biased secreted proteins. Two additional families with putative neural/developmental roles (sleep homeostasis regulator IPR050975; UPAR/Ly6 IPR031424) rounded out the M-biased signature. These functions match the biology of adult *P. ficus* males, which are short-lived, winged, nonfeeding morphs whose primary roles are mate location and reproduction. Together, these 8 enriched domains are carried by 18.2% (63 of 346) of M-biased secreted proteins.

**Table 3.** InterPro domains significantly enriched in sex-biased *P. ficus* predicted secreted proteins.

DE class	InterPro	Likely role	Category	% in class	% background	OR	BH-adj *P*
M-biased	IPR036728	Pheromone/general odorant-binding protein-domain superfamily	Chemoreception	9.25	1.61	6.21	9.2 × 10^−8^
	IPR006170	Pheromone/general odorant-binding protein	Chemoreception	7.51	1.40	5.70	6.6 × 10^−6^
	IPR000618	Insect cuticle protein	Cuticle and exoskeleton	6.36	1.19	5.62	6.1 × 10^−5^
	IPR031311	Chitin-binding type R&R consensus	Cuticle and exoskeleton	4.05	0.63	6.63	2.4 × 10^−3^
	IPR050975	Sleep homeostasis regulator	Neural/developmental	2.31	0.21	11.16	2.6 × 10^−2^
	IPR031424	UPAR/Ly6 domain-containing protein	Neural/developmental	2.60	0.35	7.58	3.4 × 10^−2^
	IPR051217	Insect cuticle structural	Cuticle and exoskeleton	2.89	0.49	6.02	3.6 × 10^−2^
	IPR050468	Larval/pupal cuticle protein	Cuticle and exoskeleton	3.18	0.63	5.16	3.6 × 10^−2^
F-biased	IPR036610	PEBP-like superfamily	PEBP-like binding	5.19	0.42	13.06	3.4 × 10^−8^
	IPR035810	PEBP protein	PEBP-like binding	3.46	0.31	11.40	6.7 × 10^−5^
	IPR018631	AAA-ATPase-like domain	AAA-ATPase	4.69	0.94	5.21	1.4 × 10^−4^

One-sided Fisher's exact test, BH corrected; only domains with adjusted *P* < 0.05 shown.

% in class, percentage of the focal M- or F-biased set carrying the domain; % background, percentage of all other tested secreted proteins carrying it; OR, odds ratio.

In contrast, only 3 InterPro domains were significantly enriched among F-biased secreted proteins (Table 3), members of just 2 families: the PEBP/PEBP-like family (IPR036610, IPR035810; OR 13.1 and 11.4) and an AAA-ATPase-like domain (IPR018631). The 3 enriched domains are carried by only 9.9% (80 of 810) of F-biased secreted proteins, a smaller fraction than the 18.2% (63 of 346) covered by the 8 M-biased domains despite F-biased proteins being more numerous. This indicates that the female feeding secretome is functionally heterogeneous and not dominated by a single conserved domain architecture. A substantial fraction of F-biased secreted proteins carries no detectable InterPro domain and likely represents novel, *P. ficus*-specific secreted proteins awaiting structural and functional characterization.

### Genomic clustering of sex-biased genes

To identify genomic regions enriched for sex-biased expression, Ps scaffolds with at least 10 tested genes (*n* = 260) were screened by Fisher’s exact test (BH-corrected). Six scaffolds showed significant enrichment (*P*_adj_ < 0.05; Fig. 4; Table 4), ranging from 145 to 824 kb in size: 5 enriched for female-biased genes (Ps350, 439 kb; Ps72, 824 kb; Ps138, 549 kb; Ps376, 569 kb; Ps743, 145 kb) and 1 for male-biased genes (Ps328, 378 kb). The most extreme cases were Ps350 (66 genes on the scaffold, 58 tested, of which 46 sex-biased: 45 F-biased, 1 M-biased; *P*_adj_ = 3.2 × 10^−6^) and Ps72 (103 genes, 81 tested, of which 37 sex-biased: 36 F-biased, 1 M-biased; *P*_adj_ = 1.4 × 10^−4^). To test whether this clustering exceeded the level expected by chance, we permuted sex-bias labels across all tested Ps genes 1,000 times and recomputed scaffold enrichment with the same procedure. The permutation null produced a mean of 0.03 significantly enriched scaffolds (range 0 to 2) for each direction, far below the observed counts (empirical *P* < 0.001). Sex-biased expression in *P. ficus* is therefore not only pervasive but also strongly segregated into discrete genomic blocks. Directional tests (separate 1-sided Fisher tests for female and male enrichment per scaffold) identified 6 additional scaffolds (130 to 554 kb) with extreme 1-sided enrichment (3 F-only: Ps233, Ps353, Ps799; 3 M-only: Ps130, Ps236, Ps259; Supplementary Table 16), all consistent with the segregation pattern (Supplementary Fig. 4).

**Fig. 4.**
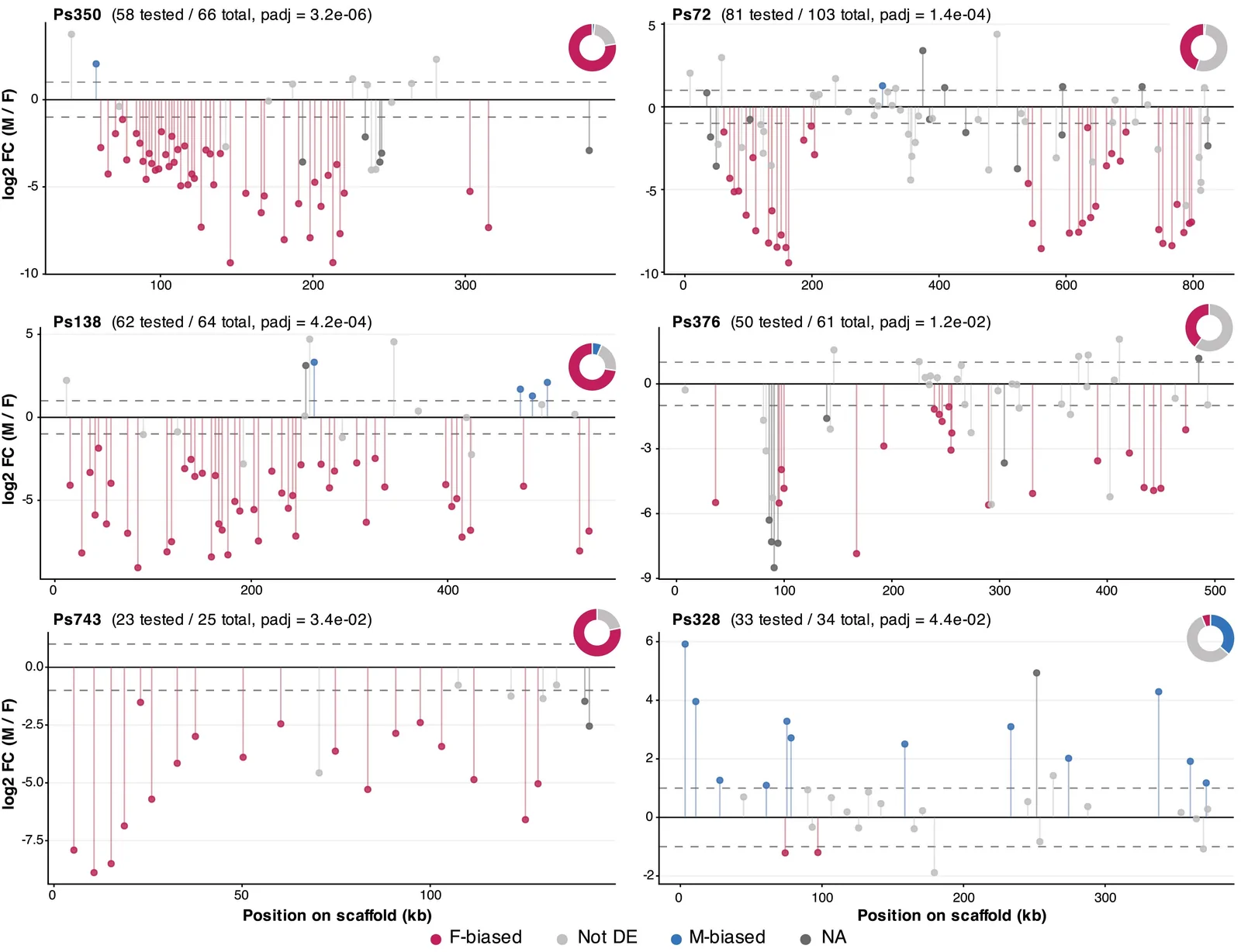
Significant sex-biased *P. ficus* scaffolds. Differential expression along the 6 Ps scaffolds that are significantly enriched for sex-biased genes (directional Fisher's exact test, BH-adjusted *P* < 0.05). Each panel shows per-gene log_2_ fold change (male/female) plotted against the gene's midpoint position on the scaffold; colors indicate DE class (F-biased, log_2_FC <−1 and *P*_adj_ < 0.05; M-biased, log_2_FC >1 and *P*_adj_ < 0.05; Not DE; or NA for genes not tested by DESeq2). Vertical segments connect each point to the zero baseline. Panel titles report the scaffold-level Fisher-test adjusted *P*-value. The donut inset in each panel summarizes the F-biased/Not DE/M-biased composition among tested genes on that scaffold.

**Table 4.** Primary-assembly scaffolds enriched for sex-biased gene expression in *P. ficus*.

Scaffold	Size (kb)	Genes	M	F	*P*_adj_	Direction
Pficus_VMBCA21f_v1.0_Ps350	439	58	1	45	3.2 × 10^−6^	F-biased
Pficus_VMBCA21f_v1.0_Ps72	824	81	1	36	1.4 × 10^−4^	F-biased
Pficus_VMBCA21f_v1.0_Ps138	549	62	4	45	4.2 × 10^−4^	F-biased
Pficus_VMBCA21f_v1.0_Ps376	569	50	0	20	1.2 × 10^−2^	F-biased
Pficus_VMBCA21f_v1.0_Ps743	145	23	0	18	3.4 × 10^−2^	F-biased
Pficus_VMBCA21f_v1.0_Ps328	378	33	12	2	4.4 × 10^−2^	M-biased

Six Ps scaffolds (≥10 DE-tested genes) showed significant enrichment for female- or male-biased genes by Fisher's exact test (BH-corrected *P*_adj_ < 0.05).

Size, scaffold length in kilobases; Genes, number of DE-tested loci on the scaffold; M and F, number of male- and female-biased genes (|log_2_FC| >1, *P*_adj_ < 0.05); Direction, dominant sex-bias category.

Inspection of gene content within the F-enriched scaffolds revealed clustered gene-family expansions, with each F-block representing a dense local concentration of a paralogous family that is also dispersed across other scaffolds. Ps350 contains a cluster of >10 PEBPs (including PEBP-like, protein D2, and protein D3 paralogs), accounting for 9 of the 18 F-biased genes contributing to the “negative regulation of ERK1/2 cascade” GO term reported above (50%), with most of the remainder on the syntenic haplotigs Hc000358F_001 and Hc000358F_003. Ps72 carries a cluster of glycosyl- and sulfo-transferases (galactose-3-O-sulfotransferases, β1,4-galactosyltransferase 7, galactosylceramide sulfotransferases) representing the largest concentration of this family in the genome (11 of 43 F-biased members), with an additional 6 paralogs on the F-only supplementary scaffold Ps799 and 7 distributed across syntenic Hc000072F haplotig fragments. Ps743 (145 kb, 14 F-biased carboxylesterase paralogs) is the densest cluster of F-biased carboxylesterases in the genome, although additional F-biased members of this family are dispersed across multiple Ps scaffolds. The single main-text M-enriched scaffold, Ps328 (34 genes on the scaffold, 33 tested, of which 14 sex-biased: 12 M-biased, 2 F-biased; *P*_adj_ = 4.4 × 10^−2^), was functionally heterogeneous and included a copia-like retrotransposon, a cytochrome P450 (CYP4g15-like), arylalkylamine N-acetyltransferase, fibroblast growth factor receptor-like, and synaptotagmin-14; the 3 additional M-only scaffolds (Ps130, Ps236, Ps259) similarly carried zinc finger paralogs, retrovirus-related Pol polyproteins, and various signaling and transmembrane components rather than a single coherent gene family.

To characterize these blocks further, we examined 3 additional properties of the enriched scaffolds: gene density, effect size, and within-block coexpression (Supplementary Table 17). F-enriched scaffolds were significantly more gene-dense than the genome-wide median (0.121 vs 0.059 genes/kb; Wilcoxon *P* = 2.5 × 10^−4^), consistent with the clustered gene-family expansions described above; M-enriched scaffolds were not significantly denser than other scaffolds (*P* = 0.18). Effect sizes were larger for F-biased genes residing within F-blocks than for F-biased genes elsewhere (median |log_2_FC| 4.93 vs 3.91; Wilcoxon *P* = 1.0 × 10^−6^); the same comparison was not significant for M-biased genes (*P* = 0.74). Within each block, the genes contributing to the sex bias were highly coexpressed across samples: the median pairwise Spearman correlation among sex-biased genes within each enriched scaffold ranged from 0.71 to 0.89, far above the null distribution generated by 500 random 20-gene sets drawn from genome-wide sex-biased genes (mean ρ = 0.10, 95th percentile 0.71; 11 of 12 enriched scaffolds significant at null *P* ≤ 0.004; Supplementary Table 18). Repeat content of enriched scaffolds did not differ significantly from the genome-wide distribution (F-blocks median 33.9% repetitive sequence, M-blocks 31.3%, other scaffolds 37.7%; Wilcoxon *P* ≥ 0.15 for both comparisons), and neither retrotransposon nor DNA-element fractions were elevated in either block class. Together, these patterns indicate that sex-biased blocks correspond to coordinately coexpressed genomic regions rather than to repetitive sequence compartments, with the F-blocks additionally enriched for clustered tandem paralogs.

## Discussion

### A reference genome for *P. ficus*

VMBCA21f is the first long-read, scaffolded, partially diploid reference genome for *P. ficus*, following an earlier short-read draft that is not publicly available (Corcoran and Mahaffee 2024). The 369.4 Mb primary assembly is 90.0% complete in genome-mode BUSCO and carries 23,489 annotated genes, with size, GC content, and repeat fraction matching k-mer estimates from 4 California samples and within the Pseudococcidae range (Li et al. 2020; Ross et al. 2024). FALCON-Unzip retained a further 127.0 Mb of haplotigs representing alternate alleles at heterozygous regions. By current standards, the VMBCA21f assembly is fragmented and not chromosome-scale. This stems largely from the input material, which was derived from a single insect whose limited high-molecular weight DNA was committed to a single PacBio CLR library, leaving too little for the HiFi or Hi-C data needed for chromosome-level contiguity. High heterozygosity and repeat content likely added to the fragmentation. CLR reads also retain higher residual error after polishing than near-error-free HiFi reads, which is reflected in a genome-mode BUSCO completeness of 90% where residual indel error left internal stop codons in roughly a tenth of complete BUSCOs and lowered protein-mode completeness.

Alignment to *P. citri* corroborates the large-scale structure of the assembly (Supplementary Fig. 1); the conservation we observe is consistent with the broad retention of chromosomal synteny recently reported across Pseudococcidae (Mongue et al. 2026). This high collinearity reflects the close relationship between the 2 species, whose divergence we date to 2.49 Ma (95% highest posterior density (HPD) 1.71 to 3.37 Ma; Fig. 1), the first molecular estimate for this split. At deeper nodes, the Pseudococcidae crown, the Phenacoccinae/Pseudococcinae split (Hardy et al. 2008; Choi and Lee 2022), dates to 69 Ma (95% HPD 55 to 84 Ma). This is younger than the genome-scale estimate of Deng et al. (2025), who dated the family to 128.1 to 93.2 Ma, and younger still relative to the 210 to 165 Ma neococcoid origin of Vea and Grimaldi (2016), consistent with Pseudococcidae being a comparatively recent radiation within neococcoids. This difference is largely one of calibration. Deng et al. (2025) placed a mid-Cretaceous fossil minimum, *Williamsicoccus megalops*, directly on the family. We excluded this fossil: it is known only from a male specimen whose placement within Pseudococcidae is unresolved, and Vea and Grimaldi (2016) likewise treated it as a floating tip rather than a node minimum. Our nearest constraint is therefore the deeper Neococcoidea node, so the 69 Ma estimate carries no direct fossil minimum and may be a lower bound.

### Predicted secreted-protein repertoire

The *P. ficus* secretome encompasses proteins released into multiple extracellular compartments, including the gut lumen during digestion and detoxification of ingested plant material, the hemolymph, and saliva injected into host tissues during stylet penetration. The salivary fraction is of particular interest for plant–insect interactions: in other phloem-feeding hemiptera, these secretions suppress sieve element occlusion, modulate plant defense signaling, and in some cases mediate virus transmission (Tsai et al. 2008; Will et al. 2013). SignalP-based screens detect the signal peptide common to all compartments and cannot separate salivary from nonsalivary secreted proteins without tissue-specific expression data, which is not yet available for *P. ficus*. We screened the *P. ficus* primary proteome with SignalP 6.0 as a starting point for the full secretome and compared it with that of *P. citri*, the closest sequenced relative. Our screen is deliberately broad: candidates are defined by a predicted signal peptide and a mature length of 30 to 500 aa, without further filtering for transmembrane or GPI-anchor signals, subcellular localization, or tissue-specific expression, and, to our knowledge, no validated, general-purpose machine-learning effector predictor exists for insects. SignalP 6.0 distinguishes signal peptides from N-terminal transmembrane helices and so limits, but does not eliminate, inclusion of membrane proteins. The set is therefore best read as a predicted secretome that includes conventional secretory and some membrane-associated proteins alongside predicted secreted proteins; because the *P. ficus* and *P. citri* secretomes were defined identically, the cross-species comparisons are unaffected by this breadth. Although both species are polyphagous, *P. citri* has a substantially broader host range than *P. ficus* (>70 vs >16 recorded plant families; Garcia-Morales et al. 2016), and the comparison can expose lineage-specific changes in the feeding-related secretome. The 2 secretomes are similar in size (2,129 vs 2,183 candidates) and share a substantial conserved core, with roughly 3-quarters of *P. ficus* and a slightly higher fraction of *P. citri* candidates falling into cross-species clusters. This shared core is dominated by small proteins with no detectable InterPro domain, together with conserved binding and recognition families (PEBP-like, odorant-binding, immunoglobulin-like, and juvenile-hormone-binding proteins), while enzyme families are present but more dispersed. Against this shared background, lineage-specific content is modest but asymmetric, and larger in *P. ficus*. The clearest lineage-specific signal is a *P. ficus* expansion of secreted UDP-glycosyltransferases, organized as 2 tandem arrays absent from *P. citri* (Fig. 2e). UGTs glycosylate lipophilic substrates, including plant secondary metabolites, are a recurring component of insect xenobiotic detoxification (Ahn et al. 2012; Meech et al. 2019); tandem UGT cluster expansions have been linked to host-plant detoxification and adaptation in other insects (Ahn et al. 2012; Wang et al. 2024). The *P. ficus* expansion is notable in occurring in the narrower-host species rather than the broad-host *P. citri*, though the relationship between UGT copy number and host breadth is not consistent across insects (Wang et al. 2024).

### A first genome-wide baseline of nucleotide diversity and markers for population monitoring

*P. ficus* is invasive in California, where prior molecular work pointed to limited genetic variation consistent with introduction from a restricted source pool (Daane et al. 2018). No published whole-genome estimates were available to our knowledge. We resequenced 4 California field individuals against the new reference to benchmark diversity, revisit the limited-variation inference, and identify genome-anchored SSR markers for vineyard-scale monitoring. Because the reference was assembled from a single laboratory-reared female, it functions here as a coordinate and annotation scaffold rather than as a source of allele-frequency information: nucleotide diversity, per-individual heterozygosity, and SSR polymorphism were all estimated from the 4 field-collected individuals and do not depend on the reference representing a wild or typical genotype. The reference individual is itself substantially heterozygous, with 127 Mb of alternate haplotigs resolved during assembly (Table 1), indicating that the colony has not been driven toward homozygosity by inbreeding or selection.

The genome-wide π of 2.06 × 10^−3^ is higher than would be expected from a severely depleted postintroduction gene pool. This is the first whole-genome estimate for California *P. ficus* and mildly complicates the inference of limited variation drawn from earlier marker-based work. Several nonexclusive interpretations are compatible with the data. The California population may have retained more standing variation than previously appreciated. Alternatively, the elevated diversity may reflect multiple or ongoing introductions rather than a single founder event: *P. ficus* continues to spread, and new distribution records have accrued since the analysis of Daane et al. (2018), so an established region such as California may receive recurrent introductions that raise diversity through admixture. A third possibility is that these 4 field individuals capture more diversity than is present across the broader California range. Distinguishing these possibilities will require denser sampling across vineyards and producing regions, ideally paired with samples from the Middle Eastern source range identified by Daane et al. (2018). Four individuals are a small sample and cannot resolve the invasion history of California *P. ficus*, which may involve a single founder event, recurrent introductions, or both; these estimates are therefore a first whole-genome benchmark rather than a population-level characterization. As the first genome-wide estimate of nucleotide diversity for *P. ficus*, this benchmark provides a reference point for future work on comparisons among geographically distinct populations, phylogeographic and demographic analyses, and characterization of diversity across the species’ native and invasive ranges.

The 132 cross-validated polymorphic SSR markers are the immediate practical output (Supplementary Table 13). Each marker is polymorphic across the 4 California field individuals and should be directly usable for vineyard-scale population monitoring, addressing a longstanding need for population-genetic tools in vine mealybug research (Daane et al. 2018).

### Pervasive, female-skewed sex-biased expression

Adult male and female *P. ficus* differ dramatically in morphology (Fig. 1) and life history. Females are large, sessile, neotenic, and feed continuously over an extended adult lifespan; males are small, winged, nonfeeding, and live a few days dedicated to mate location (Daane et al. 2012; Bain et al. 2021). Mealybugs achieve this dimorphism without sex chromosomes: sex is determined by PGE, in which males are diploid at fertilization but transcriptionally silence their paternally inherited chromosomes during early development (Bain et al. 2021; de la Filia et al. 2021). We sequenced adult male and female transcriptomes to ask 2 questions: how is sex-biased expression organized in *P. ficus*, and how much of it reflects PGE silencing itself?

About 40% of the genes were DE between adult females and males. Female-biased loci outnumber male-biased loci 1.57-fold and show 1.7-fold larger median fold changes. A comparable female skew at the level of moderately sex-biased genes has been reported in *P. citri* (Bain et al. 2021), although in that species the most extreme bias (>10-fold) is male-directed; the same study found female- and male-biased genes both enriched for core biological processes. Male-biased genes are enriched for sensory and locomotor functions (odorant binding, nervous system, muscle, ion transport, and cilium organization), and female-biased genes for core biosynthesis (translation, ribosome biogenesis, mRNA metabolism, and nuclear/nucleolar components); this functional partition (male sensory, locomotor, and structural vs female biosynthetic) parallels the categories described in *M. hirsutus* (Kohli et al. 2021), although that study identified far fewer sex-biased genes and no genome-wide expression difference between the sexes. These categories are consistent with the divergent adult biology of the 2 sexes but do not by themselves resolve whether they reflect direct sex-specific specialization or downstream consequences of upstream regulators of dimorphism, which in mealybugs operate through PGE rather than a conventional sex-determination cascade (Bain et al. 2021; de la Filia et al. 2021). They are also subject to a sampling caveat inherent to whole-body RNA-seq in an extremely dimorphic species: because adult males and females differ markedly in body organization (for example, the winged, mate-searching male with its sensory and flight apparatus vs the neotenic, sessile female), some sex-biased signals, particularly sensory categories such as odorant binding, may partly reflect the differing relative contributions of tissues to the whole-body RNA pool rather than differences in per-cell expression. Two features of the design further limit these inferences. The transcriptome derives from a long-term laboratory colony, and because laboratory adaptation can shift expression, the absolute magnitude of sex bias may differ in field populations, although the qualitative pattern of pervasive, female-skewed, clustered expression is unlikely to be a rearing artifact. Because the assembly is partially diploid, with heterozygous regions retained as separate haplotigs, a subset of loci is represented twice and reads at these loci are split between haplotype copies; this makes their gene-level counts conservative and reduces power to detect subtle differences, so the sex-biased set we report is likely an underestimate rather than an overestimate.

Both sex-biased gene sets are enriched for predicted secretion relative to unbiased genes, consistent with secretion being central to the adult biology of both sexes, but the 2 secretomes differ in functional composition and evolutionary origin. The female-biased enrichment fits the continuous feeding of the sessile female, and the male-biased enrichment is dominated by odorant-binding, cuticle, and chitin-binding proteins, fitting the short-lived, dispersing, mate-locating male (Corcoran and Mahaffee 2024). More informative than the enrichment itself is the evolutionary origin of the 2 sets. The male-biased secretome is drawn preferentially from secreted proteins with 1:1 orthologs in *P. citri*, whereas the female-biased secretome spans the broader, lineage-specific secreted-protein pool and is functionally heterogeneous, with few enriched domains over a large candidate set and many members lacking any detectable InterPro signature. One interpretation is lineage-specific selection acting on the feeding-related secretome, with the female repertoire diversifying on the *P. ficus* lineage while the male secretome remains anchored in a conserved, shared core, consistent with the faster evolution and lower orthology of female-biased genes reported in *P. citri* (Mongue et al. 2025) and with the lineage-specific expansion of salivary effectors documented in aphids (Boulain et al. 2018). We emphasize that these functional attributions, the detoxification role of the UGT expansion, the feeding role of the female-biased secretome, and the reproductive role of the male-biased secretome, are inferred from domain annotation, sequence homology, and sex-biased expression, and none has been tested directly; they are hypotheses for functional and tissue-specific follow-up.

PGE silencing offers a candidate mechanism for the female skew documented above. If paternal alleles in males were uniformly silenced without compensation, male output would be globally halved relative to diploid females, producing a genome-wide log_2_FC near −1. Our data do not show this (Fig. 3c). The Ps median is −0.33 (mode near zero), meaning the median gene expresses about 80% of female level rather than 50%. This matches allele-specific RNA-seq in *P. citri* and *P. citri* × *P. ficus* hybrid males, where paternal silencing in soma is strong but incomplete, with up to ∼70% of somatically expressed genes retaining some paternal contribution even as maternal alleles dominate globally (only ∼20% of genes showing any paternal contribution; de la Filia et al. 2021). Bain et al. (2021) proposed female hypermethylation in *P. citri* as a possible mechanism of ploidy compensation. Somatic paternal silencing in *P. ficus* therefore appears partial and is not on its own sufficient to explain the female-skewed sex bias we observe.

### Genomic clustering of female-biased expression

The most striking finding from the expression analysis is that female-biased expression is not dispersed across the genome but concentrated in a small number of discrete scaffold-level blocks. The same is not true to nearly the same degree for male-biased expression. Permutation of sex-bias labels confirms that this degree of clustering is far beyond chance (Fig. 4; Table 4), so the blocks are a real feature of genome organization rather than a sampling artifact of a few gene-rich scaffolds. What makes these female blocks distinctive is not simply that they carry many female-biased genes, but that they share 3 properties that together point to coordinate regulation. They are unusually gene-dense; they each harbor a local expansion of a single gene family: PEBPs on Ps350, glycosyl- and sulfo-transferases on Ps72, carboxylesterases on Ps743; and the female-biased genes within a block are tightly coexpressed across samples, far more so than female-biased genes drawn at random from the genome. Female-biased genes inside these blocks are also more strongly biased than female-biased genes elsewhere, and the blocks are not enriched for repetitive sequence. The blocks therefore could be interpreted as coordinately regulated genomic units rather than incidental clusters of colocated paralogs. We are unaware of previous reports of female-restricted coordinate regulation of clustered tandem paralogs on this scale in a hemipteran that lacks sex chromosomes. We draw this as a contrast in genomic organization rather than a mechanistic claim: in aphids, sex-biased gene enrichment is concentrated on the X chromosome, reflecting the evolutionary dynamics of the X under X0 sex determination (Jaquiéry et al. 2013; Li, Zhang et al. 2020), a genomic substrate absent in *P. ficus*, where sex is determined through PGE without heteromorphic sex chromosomes (Kol-Maimon et al. 2014; Bain et al. 2021; Mongue et al. 2026). Our data establish the existence of clustered, female-biased expression but do not establish that this organization is a consequence of PGE or of the absence of sex chromosomes; tandem duplication, shared cis-regulatory elements, chromatin organization, and lineage-specific gene-family expansion could each generate such clustering independently of the sex-determination system.

The biological roles of these expansions remain to be studied. PEBPs on Ps350 belong to a family with established roles in MAPK/ERK signaling (Yeung et al. 1999) and account for the GO enrichment for negative regulation of the ERK1/2 cascade among female-biased genes. The Ps72 glycosyl- and sulfo-transferase cluster is plausibly involved in glycan modification of cuticular or secreted proteins. The Ps743 carboxylesterase cluster is of immediate applied interest: carboxylesterases are recurrent substrates for metabolic insecticide resistance in hemipteran pests, often via gene amplification and overexpression, as in the amplified E4/FE4 esterases of *Myzus persicae* (Bass et al. 2014) and esterase-mediated organophosphate resistance in *Nilaparvata lugens* (Small and Hemingway 2000). A female-specific, coordinately expressed esterase expansion is therefore a plausible substrate for resistance evolution under the insecticide-heavy management typical of vine mealybug (Mansour et al. 2018; Daane et al. 2020).

### Male-biased expression clusters without gene-family expansion

Male-biased expression is also clustered, but the M-enriched scaffolds differ from F-enriched ones in 3 respects: they are not gene-dense above the genome background, they lack obvious tandem family expansions and instead contain heterogeneous gene content (zinc finger paralogs, retrovirus-related Pol polyproteins, a CYP4g15-like P450, signaling components), and the effect sizes of their male-biased genes are statistically indistinguishable from male-biased genes elsewhere. What they share with F-blocks is strong within-block coexpression, consistent with coordinate regulation despite heterogeneous gene content. The 2,862 male-biased genes cannot be a passive consequence of uniform paternal-genome silencing. Under PGE, silencing would produce a global deficit of male transcription, not active large-effect male upregulation. These genes are actively upregulated in males despite the halved paternal-genome dose, and the M-enriched scaffolds represent regions where male-specific regulatory programs override the silencing baseline. Whether they correspond to regions of male-specific transcriptional activation, regions where paternal alleles preferentially escape silencing, or both, cannot be resolved with bulk RNA-seq.

The asymmetry between sexes (more female-biased genes, larger female-biased effects, gene-rich tandem-expanded female blocks against heterogeneous modest-effect male blocks) fits a regulatory architecture in which the male transcriptome is more constrained than the female one. Mongue et al. (2025) reported lower variation and slower sequence evolution of male-biased genes in *P. citri*, alongside signatures of stronger adaptation; our data extend that to *P. ficus* and add a spatial dimension, in which female-biased gene evolution in mealybugs is proceeding preferentially through tandem duplication and coordinate regulation of clustered paralogs.

In summary, sex-biased expression in *P. ficus* is spatially structured, and the structure differs by sex. Female-biased genes cluster into gene-dense blocks of coexpressed tandem paralogs; male-biased genes cluster into coexpressed but compositionally heterogeneous regions without family expansion. These are descriptive genomic patterns from a single bulk RNA-seq comparison of 3 females and 3 males; their regulatory basis, their relationship to PGE, and any functional consequences remain to be tested directly.

## Supplementary Material

jkag235_Supplementary_Data

## Data Availability

Raw sequencing reads and the genome assembly/annotations were deposited at NCBI under BioProject PRJNA1481362, PRJNA1482103 (primary scaffolds), and PRJNA1482104 (haplotigs). PASS-filtered SNP and indel VCFs together with all assembly and annotation files (fasta and gff) are available at Zenodo (10.5281/zenodo.20854017; https://zenodo.org/records/21039194). A dedicated genome browser is available at www.grapegenomics.com (https://www.grapegenomics.com/pages/Pficus/). Analysis code is available at https://github.com/CantuLab/VMB_genome_analyses. Supplemental material available at G3 online.
